# Mediterranean diet adherence and nutritional literacy: an observational cross-sectional study of the reality of university students in a COVID-19 pandemic context

**DOI:** 10.1136/bmjnph-2023-000659

**Published:** 2023-12-19

**Authors:** Filipa Abreu, Ana Hernando, Luís Filipe Goulão, Alexandra Marques Pinto, Amélia Branco, Ana Cerqueira, Cecília Galvão, Fábio Botelho Guedes, Maria Rosário Bronze, Wanda Viegas, Margarida Gaspar Matos, Joana Sousa

**Affiliations:** 1 Universidade de Lisboa Faculdade de Medicina, Lisboa, Portugal; 2 Universidade de Lisboa Instituto Superior de Agronomia, Lisboa, Portugal; 3 Universidade de Lisboa Faculdade de Psicologia, Lisboa, Portugal; 4 Universidade de Lisboa Instituto Superior de Economia e Gestão, Lisboa, Portugal; 5 Universidade de Lisboa Instituto de Saúde Ambiental, Lisboa, Portugal; 6 Universidade de Lisboa Faculdade de Motricidade Humana, Cruz Quebrada, Portugal; 7 Universidade de Lisboa Instituto de Educação, Lisboa, Portugal; 8 Universidade de Lisboa Faculdade de Farmácia, Lisboa, Portugal; 9 Instituto de Biologia Experimental e Tecnológica, Oeiras, Portugal; 10 Instituto Universitário de Ciências Psicológicas, Sociais e da Vida, Lisboa, Portugal

**Keywords:** COVID-19, Dietary patterns

## Abstract

**Aim:**

To evaluate the adherence to the Mediterranean diet (MD) and the level of nutritional literacy (NL) among university students from different academic fields of study, within the context of the COVID-19 pandemic.

**Methods:**

A total of 1114 first-year undergraduate students at the University of Lisbon, Portugal, were included in this study. A self-administered online questionnaire was applied that included questions regarding sociodemographic information, the MD measured by the PREDIMED questionnaire (PREvención con DIeta MEDiterránea) and NL assessed using the Newest Vital Sign questionnaire.

**Results:**

The average PREDIMED score revealed a low adherence (6.79±2.14 points) to the MD. Notably, students in the Social Sciences and Humanities academic fields showed the highest level of adherence (U=21 071; p<0.05). Within the Health field, there was a greater prevalence of dietary behaviours aligned with the MD, contributing to higher overall adherence scores. Furthermore, 84.1% of the participants demonstrated adequate NL. Interestingly, students in the Exact Sciences and Engineering field demonstrated the highest levels of NL (5.07±1.19), particularly in questions involving mathematical reasoning.

**Conclusions:**

Our findings suggest that university students in Lisbon do not follow a MD and are far from the recommendations of this dietary pattern. While most participants showed adequate NL, it is essential to highlight the link between knowledge and application to daily practice. Despite positive literacy levels, there remains a deficit in translating this knowledge into correct dietary practices.

**WHAT IS ALREADY KNOWN ON THIS TOPIC**

- Adherence to the Mediterranean diet (MD) is low among university students, and remarkably, about half of the Portuguese population have inadequate general health literacy levels, slightly below the average compared with other European countries.

**WHAT THIS STUDY ADDS**

- Adherence to the MD and nutritional literacy (NL) are significantly different across the different academic fields of study. There seems to be a positive trend between NL and MD adherence. Also, both parents’ educational levels exhibit a positive association with MD adherence and the mother’s educational level with NL.

**HOW THIS STUDY MIGHT AFFECT RESEARCH, PRACTICE OR POLICY**

- Findings hold the potential to support and drive the development of public and community health initiatives in university settings.

## Introduction

For most young adults the beginning of academic life is an important milestone for independence and responsibility, and for many, it means moving abroad, away from home and family to live alone. This implies a greater responsibility and autonomy when choosing meals.

This period can be particularly vulnerable to making incorrect food choices, potentially leading to nutritional deficiencies, weight gain or even weight loss, which can have implications for overall health.^1 2^ Previous studies have demonstrated that university students frequently exhibit dietary patterns that diverge from what is commonly regarded as healthy. These patterns are characterised by reduced intake of fruits and vegetables and increased consumption of energy-dense foods.^1 2^

The COVID-19 pandemic is an important context as many studies have shown this period was particularly vulnerable to poor nutritional behaviours and reduced physical activity, resulting in unhealthy weight.^3^

The pandemic started in Portugal in March 2020, leading to a lockdown that lasted until May 2020. The severity of restrictions varied across countries, with Portugal initially experiencing a higher number of cases than the European Union average.^4 5^ This period was particularly challenging for the Portuguese due to the restrictions implied in the country. The Directorate-General of Health of Portugal (DGS) conducted a national study to evaluate the dietary behaviours of the population during the first isolation period (April to May 2020). In Portugal, there was an increase in the consumption of water, sweet snacks, fruits and vegetables. Conversely, there was a decrease in the consumption of takeaway meals, pre-prepared meals, soft drinks and alcoholic drinks.^6^

The Mediterranean diet (MD) is one of the world’s healthiest dietary patterns.^7^ It is distinguished by being low in saturated fat and animal protein, high in antioxidants, fibre and monounsaturated fat, as well as having an adequate omega-6 to omega-3 fatty acid ratio.^8^

The beneficial effects of the MD in preventing various types of chronic diseases including cancer, cardiovascular diseases and diabetes mellitus are well established.^9^ For these reasons, the WHO considers the MD as a health promoter; it has been distinguished by the United Nations Educational, Scientific and Cultural Organization as a World Heritage of Humanity.^10^ Nevertheless, the nutritional impact originated from the cultural globalisation of food markets and changes in work patterns have contributed to a change in eating habits, resulting in a decreased adherence to these traditional dietary patterns, such as the MD.^11^

In Portugal, adherence to the MD decreased between 2017 and 2019, accompanied by an increase in the consumption of foods that are not characteristic of this dietary pattern.^12^

For a positive adherence to a healthy eating pattern, consumers’ level of nutritional literacy (NL) is fundamental. NL is defined as the individual’s ability to access, process and understand nutritional information and its impact on health.^13^ While food literacy (FL) also involves nutritional information, it includes other aspects like culture, environment, food identity and eating behaviour.^14^

Almost half of the Portuguese population have inadequate general health literacy levels,^14 15^ being slightly below the average compared with other European countries. Although there are no data available on NL or FL in Portugal, considering the low levels observed for general health literacy of the population, it is assumed that their NL does not differ substantially. A pilot study based on Portuguese adults showed that more than half (65.2%) had a good level of NL^12^; however, 27.8% of the study population were studying/working in the area of Health, limiting the validity of results. In fact, the authors acknowledge that these NL levels were conditioned by having upper educational qualifications, family members trained in the field of nutrition and by the fact of studying/working in the Health area, raising concerns about the representativity and generalisability of these results to the general population.

To evaluate NL, there are several validated questionnaires, among them is the Newest Vital Sign (NVS) questionnaire. The NVS is one of the most widely used health literacy screening instruments and has been extensively used in other studies to evaluate NL.^16^ To administer the NVS, individuals were presented with a nutritional label from a container of ice cream and asked six questions about the label.

According to the national guidelines for healthy eating implemented during the pandemic, it was found that those who were unaware of these guidelines were most likely to adopt an unhealthy eating pattern.^6^ This result is not surprising, instead, highlights the importance of NL and the value of developing information and campaigns to promote healthy dietary choices, ensuring the population is well informed to make healthy decisions.

The present study aims to analyse and characterise the adherence to the MD and the level of NL among university students during the COVID-19 pandemic, among different academic fields of study, as well as investigate any potential correlations between NL and adherence to the MD.

## Methods

This research was carried out as an observational cross-sectional study under the HOUSE Project from the Colégio F3—Food, Farming, Forestry, from the University of Lisbon. The project’s main objective was to explore the health and lifestyle characteristics of first-year students at the University of Lisbon, Portugal.

The survey was sent to students through their institutional emails. The questionnaire was applied online and consisted of six dimensions: (1) health and well-being, (2) use of psychotropic and potentially addictive substances, (3) physical activity, (4) eating habits, (5) physical and emotional issues and (6) NL/FL and knowledge. The questions used in the present study were based on two specific dimensions: eating habits, assessed using the Portuguese version of the PREDIMED questionnaire (PREvención con DIeta MEDiterránea) and NL, evaluated using NVS the questionnaire, which has also been previously validated.

The inclusion criteria for this study were to be enrolled in the first year at the University of Lisbon and to be above 18 years of age.

The data was analysed according to the area of study; we included five academic fields: (1) Arts, (2) Exact Sciences and Engineering, (3) Natural Sciences, (4) Health and (5) Social Sciences and Humanities; online supplemental appendix 1 displays the distribution of faculties by academic field of study. To assess adherence to the MD, the validated Portuguese version of the PREDIMED was used,^17^ scoring between 0 and 14. Adherence was classified as good if the score was ≥10 and poor if the score was <10. NL was assessed using the NVS questionnaire, also validated for the Portuguese population.^18^

10.1136/bmjnph-2023-000659.supp1 Supplementary data

Data analysis was performed using the software IBM-SPSS V.25.0 (SPSS). The Kolmogorov–Smirnov test was used to determine the normality of data distributions. Continuous variables are expressed as mean±SD, and categorical variables are expressed as percentages.

For comparisons between groups, the Mann-Whitney test was used to compare MD adherence and NL between different genders and academic fields of study. The Kruskal-Wallis test was used to compare overall PREDIMED and NVS scores according to the different academic fields of study. The Spearman correlation coefficient was used to evaluate the association between variables with non-normal distribution. The χ^2^ test was used to study nominal variables and the Monte Carlo simulation χ^2^ test was used whenever the applicability assumptions of the χ^2^ test were not verified. Finally, the eta coefficient test was used to determine the strength of association between each question in the PREDIMED questionnaire and the overall PREDIMED score. Significance was fixed at p<0.05.

## Results

The questionnaire was sent to a total of 47 005 emails, out of which only 7895 were first-year undergraduates. The final sample included 1114 students, indicating a participation rate of approximately 17.4%. Among the respondents, 754 (67.7%) were female, with ages ranging from 18 to 30 years (mean (M)=19.10 years old; SD=1.72). The majority were Portuguese (93.1%, n=1037), and half were from the city of Lisbon, Portugal (51.8%, n=577). Overall, the respondents started higher education in the year 2020/2021 (88.9%; n=990), and the most represented academic field of study was Exact Sciences and Engineering (24.2%; n=270); these results are represented in table 1. It is important to note that the data collected did not follow a normal distribution.

**Table 1:** Participants’ characterisation (n=1114)

Age (years)	19.10±1.72
Gender	
Female	67.7 (754)
Male	31.2 (348)
Rather not answer	1.1 (12)
Student’s origin	
Lisbon District	51.8 (577)
Another Portuguese district	46.0 (512)
Erasmus	0.1 (1)
Portuguese-speaking African countries (PALOP)+Timor International Scholarship	0.3 (3)
Brasil International Scholarship	0.6 (7)
Another international mobility programme	1.3 (14)
Academic fields of study	
Health	22.1 (246)
Social Sciences and Humanities	22.5 (251)
Arts	7.3 (81)
Natural Sciences	23.9 (266)
Exact Sciences and Engineering	24.2 (270)
Academic qualifications	
Father	
Never studied	0.3 (3)
Elementary school	4.9 (55)
Middle school	19.3 (215)
High school	29.9 (333)
Higher education	43.5 (485)
Don’t know	2.1 (23)
Mother	
Never studied	0.2 (2)
Elementary school	2.9 (32)
Middle school	12.7 (142)
High school	26.3 (293)
Higher education	57.3 (638)
Don’t know	0.6 (7)
Mediterranean Diet adherence (points)	6.79±2.14
Good adherence	10.5 (101)
Low adherence	89.5 (861)
Nutritional literacy (points)	4.77±1.44
Limited literacy	4.9 (25)
Possibility of limited literacy	11.1 (57)
Adequate literacy	84.1 (433)

Results are expressed as % (number of individuals) or mean±SD.

MD adherence, measured by the total score of the PREDIMED, showed a mean score of 6.79±2.14 points with 89.5% (n=861), representing an overall low adherence to the MD within this sample. Regarding the main type of fat used when cooking, 88.9% (n=983) reported using olive oil; however, only 6.8% (n=75) used above the recommendation (≥4 tablespoons/day). Regarding meat consumption, it was verified that half (55.5%; n=613) preferred white meats over red meats; however, only 32.9% (n=359) consumed less than one serving of red meats (100 g) per day. For fish consumption, 50.1% (550) of participants consumed ≥3 servings of fish per week. At last, vegetable intake showed a higher adherence, with 56.2%; n=620 of the population consuming ≥2 servings per day, and for fruits, a different pattern was observed as only 23.2% (n=256) consumed ≥3 servings per day.

Statistically significant differences were observed between MD adherence and the academic field of study (U=21 071; p=0014), with the Social Sciences and Humanities showing the highest mean MD adherence (7.25±2.13). No significant difference was found between gender and MD adherence. However, when analysing the answers for the individual questions of the PREDIMED, significant differences between genders were observed, suggesting that the female gender tends to have better eating behaviours; these observations are shown in table 2; particularly, regarding the daily consumption of olive oil (p<0.05; 95% CI 0.009, 0.013), red meats (p<0.001; 95% CI 0.000, 0.000), butter, margarine or cream (p<0.001; 95% CI 0.000, 0.000), sugary or carbonated beverages (p<0.05; 95% CI 0.010, 0.014), consumption of fish or seafood (p<0.01; 95% CI 0.008, 0.011) and preference for white meats over red meats (p<0.001; 95% CI 0.000, 0.000).

**Table 2:** Differences between genders for the responses to the PREDIMED (PREvención con DIeta MEDiterránea) questionnaire

	Total of correct answers % (n)	F% (n)	M% (n)	P value
Do you use olive oil as main culinary fat? (Yes) (n=1106)	88.9 (983)	68.9 (677)	30.0 (295)	0.155
How much olive oil do you consume in a given day? (≥4 tbsp) (n=1099)	6.8 (75)	68.0 (51)	30.7 (23)	**0.049**
How many vegetable servings do you consume per day? (≥2) (n=1103)	56.2 (650)	70.0 (433)	29.4 (182)	0.088
How many fruit units do you consume per day? (≥3 pieces) (n=1104)	23.2 (256)	74.5 (190)	25.5 (65)	**0.011**
How many servings of red meat, hamburger or meat products do you consume per day? A full serving is 100–150 g (<1 portion) (n=1092)	32.9 (359)	77.9 (279)	20.7 (74)	**0.000**
How many servings (12 g) of butter, margarine or cream do you consume per day? (<1 portion) (n=1088)	55.1 (600)	72.2 (434)	26.1 (157)	**0.000**
How much sweetened or carbonated beverages do you drink per day? (<1 per day) (n=1051)	81.3 (854)	68.7 (587)	30.6 (261)	**0.012**
Do you drink wine? How much do you drink per week? (≥7 cups) (n=1002)	0.8 (8)	25.0 (2)	75.0 (6)	0.103
How many servings (150 g) of legumes do you consume per week? (≥3) (n=1093)	53.8 (587)	69.3 (407)	29.1 (171)	0.102
How many servings of fish/seafood do you consume per week? (100–150 g of fish, 200 g of seafood) (≥3) (n=1095)	50.1 (550)	66.9 (368)	32.9 (181)	**0.010**
How many times per week do you consume commercial sweets or pastries, such as cakes, cookies, biscuits or custard? (<3 times) (n=1093)	59.3 (648)	68.5 (444)	30.1 (195)	0.370
How many servings of nuts (including peanuts) do you consume per week? (1 serving: 30 g) (≥3) (n=1079)	21.3 (230)	65.7 (151)	33.9 (78)	0.388
Do you preferentially consume chicken, turkey or rabbit meat instead of veal, pork, hamburger or sausage? (Yes) (n=1104)	55.5 (613)	74.2 (455)	25.3 (155)	**0.000**
How many times per week do you consume vegetables, pasta, rice or other dishes seasoned with sofrito (sauce made with tomato and onion, leek or garlic and simmered with olive oil)? (≥2) (n=1102)	88.6 (976)	67.6 (660)	31.3 (305)	1.000

Bold denotes statistically significant differences (p<0.05).

The distribution of the answers on the PREDIMED varied across the different academic fields (table 3). Significant differences were observed in the consumption of fruits (p<0.05; 95% CI 0.017, 0.022), red meats (p<0.01; 95% CI 0.036, 0.043) and white meats (p<0.05; 95% CI 0.019, 0.025), with Health being the academic field with the highest adherence to these aspects. Differences were also found in the consumption of sugar-sweetened and carbonated beverages (p<0.005; 95% CI 0.002, 0.004), and the weekly intake of fish and seafood (p<0.001; 95% CI 0.000, 0.000), with students from the field of Exact Sciences and Engineering showing a higher adherence to these aspects.

**Table 3:** Results of the PREDIMED (PREvención con DIeta MEDiterránea) questionnaire by academic field of study

	Health% (n)	Social Sciences and Humanities% (n)	Arts% (n)	Natural Sciences% (n)	Exact Sciences and Engineering% (n)	P value
Do you use olive oil as main culinary fat? (Yes) (n=1106)	22.6 (222)	22.6 (222)	7.0 (69)	22.9 (225)	24.9 (245)	0.241
How much olive oil do you consume in a given day? (≥4 tbsp) (n=1099)	24.0 (18)	24.0 (18)	2.7 (2)	26.7 (20)	22.7 (17)	0.584
How many vegetable servings do you consume per day? (≥2) (n=1103)	25.2 (156)	21.0 (130)	6.5 (40)	22.6 (140)	24.7 (153)	0.066
How many fruit units do you consume per day? (≥3 pieces) (n=1104)	28.2 (72)	23.1 (59)	5.1 (13)	18.4 (47)	25.1 (64)	**0.019**
How many servings of red meat, hamburger or meat products do you consume per day? A full serving is 100–150 g (<1 portion) (n=1092)	23.5 (84)	21.8 (78)	10.6 (38)	21.8 (78)	22.3 (80)	**0.040**
How many servings (12 g) of butter, margarine or cream do you consume per day? (<1 portion) (n=1088)	23.6 (142)	21.6 (130)	8.0 (48)	22.1 (133)	24.6 (148)	0.351
How much sweetened or carbonated beverages do you drink per day? (<1 per day) (n=1051)	23.3 (199)	21.3 (182)	7.5 (64)	22.1 (189)	25.8 (220)	**0.003**
Do you drink wine? How much do you drink per week? (≥7 cups) (n=1002)	0 (0)	0 (0)	25.0 (2)	37.5 (3)	37.5 (3)	0.069
How many servings (150 g) of legumes do you consume per week? (≥3) (n=1093)	21.0 (123)	23.7 (139)	8.7 (51)	23.7 (139)	23.0 (135)	0.201
How many servings of fish/seafood do you consume per week? (100–150 g of fish, 200 g of seafood) (≥3) (n=1095)	25.8 (142)	19.5 (107)	4.9 (27)	22.9 (126)	26.9 (148)	**0.000**
How many times per week do you consume commercial sweets or pastries, such as cakes, cookies, biscuits or custard? (<3 times) (n=1093)	23.8 (154)	20.8 (135)	7.9 (51)	22.5 (146)	25.0 (162)	0.210
How many servings of nuts (including peanuts) do you consume per week? (1 serving: 30 g) (≥3) (n=1079)	26.5 (61)	18.7 (43)	5.7 (13)	21.3 (49)	27.8 (64)	0.111
Do you preferentially consume chicken, turkey or rabbit meat instead of veal, pork, hamburger or sausage? (Yes) (n=1104)	25.6 (157)	21.7 (133)	6.9 (42)	24.1 (148)	21.7 (133)	**0.022**
How many times per week do you consume vegetables, pasta, rice or other dishes seasoned with sofrito (sauce made with tomato and onion, leek or garlic and simmered with olive oil)? (≥2) (n=1102)	21.5 (210)	22.7 (222)	7.1 (69)	23.9 (233)	24.8 (242)	0.329
Final score (mean±SD)	6.56±2.08	7.25±2.13	6.74±1.91	6.46±2.12	6.91±2.20	

Bold denotes statistically significant differences (p<0.05).

As displayed in table 4, in the Health field, the questions with the biggest strength in the final score were the ones that evaluated the daily consumption of portions of red meats (eta=0.455) and consumption of nuts (eta=0.442). In the fields of Social Sciences and Humanities, Exact Sciences and Engineering, and Natural Sciences, the strongest association was found in the daily fruit consumption (eta=0.419; 0.523; 0.289, respectively); and in the Arts field, it was the weekly consumption of fish/seafood (eta=0.409). An adequate level of NL was found in 84.1% (n=433) of respondents but significant differences were observed across the different academic fields of study for some of the questions (table 5). The students from the field of Exact Sciences and Engineering showed greater knowledge on the questions that required more mathematical thinking, such as ‘If you eat the total package, how many calories do you consume?’ (p<0.001; 95% CI 0.000, 0. 000), or ‘if you can eat 60g of carbohydrates in an intermediate meal, how much ice cream can you eat?’ (p<0.001; 95% CI 0.000, 0.000), and also on the question associated with the interpretation of the ingredient list and the potential allergenicity of the consumption of that food (p<0.001; 95% CI 0.000, 0.000). No correlation was observed between MD adherence and NL levels, in none of the academic fields, as displayed in table 6. However, there were significant variations in both MD adherence and NL among the various fields of study (p=0.002 and p<0.001, respectively), as indicated in table 7.

**Table 4:** Eta coefficient to the correlation between the PREDIMED (PREvención con DIeta MEDiterránea) questions and the final score by *academic field of study*

	Health	Social Sciences and Humanities	Arts	Natural Sciences	Exact Sciences and Engineering
Do you use olive oil as main culinary fat? (Yes) (n=1106)	0.120	0.094	0.033	0.115	0.118
How much olive oil do you consume in a given day? (≥4 tbsp) (n=1099)	0.141	0.008	0.053	0.273	0.304
How many vegetable servings do you consume per day? (≥2) (n=1103)	0.307	0.269	0.313	0.243	0.305
How many fruit units do you consume per day? (≥3 pieces) (n=1104)	0.384	**0.419**	0.006	**0.289**	**0.523**
How many servings of red meat, hamburger or meat products do you consume per day? A full serving is 100–150 g (<1 portion) (n=1092)	**0.455**	0.211	0.201	0.244	0.349
How many servings (12 g) of butter, margarine or cream do you consume per day? (<1 portion) (n=1088)	0.276	0.246	0.263	0.145	0.214
How much sweetened or carbonated beverages do you drink per day? (<1 per day) (n=1051)	0.160	0.136	0.120	0.148	0.159
Do you drink wine? How much do you drink per week? (≥7 cups) (n=1002)	–	–	0.053	0.031	0.066
How many servings (150 g) of legumes do you consume per week? (≥3) (n=1093)	0.204	0.175	0.233	0.264	0.227
How many servings of fish/seafood do you consume per week? (100–150 g of fish, 200 g of seafood) (≥3) (n=1095)	0.246	0.282	**0.409**	0.209	0.190
How many times per week do you consume commercial sweets or pastries, such as cakes, cookies, biscuits or custard? (<3 times) (n=1093)	0.258	0.157	0.240	0.154	0.144
How many servings of nuts (including peanuts) do you consume per week? (1 serving: 30 g) (≥3) (n=1079)	**0.442**	0.355	0.287	0.006	0.340
Do you preferentially consume chicken, turkey or rabbit meat instead of veal, pork, hamburger or sausage? (Yes) (n=1104)	0.113	0.147	0.193	0.203	0.148
How many times per week do you consume vegetables, pasta, rice or other dishes seasoned with sofrito (sauce made with tomato and onion, leek or garlic and simmered with olive oil)? (≥2) (n=1102)	0.004	0.016	0.112	0.041	0.082

Bold denotes statistically significant differences (p<0.05).

**Table 5:** Results of the NVS questionnaire by academic field of study

	Health % (n)	Social Sciences and Humanities % (n)	Arts % (n)	Natural Sciences % (n)	Exact Sciences and Engineering % (n)	P value
1. If you eat the entire container, how many calories will you consume? (1000) (n=867)	24.6 (158)	20.6 (132)	7.2 (46)	18.4 (118)	29.2 (187)	**0.000**
2. If you are allowed to consume 60 g of carbohydrates for snack, how much ice cream could you have? (1 cup or half the container) (n=1114)	25.8 (118)	17.5 (80)	5.9 (27)	19.7 (90)	31.2 (143)	**0.000**
3. Your doctor advises you to reduce the amount of saturated fat in your diet. You usually have 42 g of saturated fat each day, which includes one serving of ice cream. If you stop eating ice cream, how many grams of saturated fat would you be consuming each day? (33) (n=580)	25.5 (116)	19.1 (87)	5.7 (26)	20.0 (91)	29.7 (135)	0.109
4. If you usually consume 2500 calories in a day, what percentage of your daily value of calories will you be consuming if you eat one serving? (10%) (n=575)	23.2 (92)	18.4 (73)	6.8 (279)	19.4 (77)	32.2 (128)	**0.002**
5. Pretend that you are allergic to the following substances: penicillin, peanuts, latex gloves and bee stings. Is it safe for you to eat this ice cream? (No) (n=849)	23.7 (168)	21.7 (154)	8.0 (57)	20.1 (143)	26.5 (188)	**0.000**
6. If the patient responds ‘no’ to question (5): why not? Because it has peanut oil (n=662)	25.6 (160)	21.8 (136)	7.5 (47)	19.0 (119)	26.1 (163)	0.133
Final score (mean±SD)	5.02±1.24	4.64±1.42	4.93±1.08	4.16±1.83	5.07±1.19	

Bold denotes statistically significant differences (p<0.05).

NVS, Newest Vital Sign.

**Table 6:** Bivariate analysis of correlation between PREDIMED score (PREvención con DIeta MEDiterránea) and NVS score by academic field of study

	Spearman correlation	P value
Health	−0.022	0.821
Social Sciences and Humanities	0.089	0.416
Arts	0.342	0.081
Natural Sciences	0.035	0.736
Exact Sciences and Engineering	0.134	0.118

NVS, Newest Vital Sign.

**Table 7:** Kruskal-Wallis test for PREDIMED score (PREvención con DIeta MEDiterránea) and NVS score by academic field of study

	Field of study	n	H	P value
PREDIMED score	Health	213	16.731	**0.002**
	Social Sciences and Humanities	214		
	Arts	69		
	Natural Sciences	225		
	Exact Sciences and Engineering	241		
NVS score	Health	124	23.292	**<0.001**
	Social Sciences and Humanities	97		
	Arts	30		
	Natural Sciences	112		
	Exact Sciences and Engineering	152		

Bold denotes statistically significant differences (p<0.05).

NVS, Newest Vital Sign.

A positive correlation was seen between the mother’s level of education and NL (r=0.088, p=0.047); however, the same was not observed between the father’s educational level and NL. Likewise, as shown in table 8, both the mother and father’s educational levels were positively correlated with MD adherence (r=0.069, p=0.032 and r=0.083, p=0.010, respectively).

**Table 8:** Spearman correlation between MD adherence and NL with the mother and father’s educational levels

	Mother’s education	Father’s education	NL
MD adherence	r=0.069 **P=0.032**	r=0.083 **P=0.010**	r=0.860 P=0.067
NL	r=0.088 **P=0.047**	r=0.083 P=0.060	

Bold denotes statistically significant differences (p<0.05).

MD, Mediterranean diet; NL, nutritional literacy.

## Discussion

Regarding MD adherence, 89.5% (n=861) of participants showed a low adherence with a mean score of less than 7 points. These results are in line with other studies performed in different countries. A study based on Lebanese university students showed a mean of 8.0±2.2 points for MD adherence,^19^ while another study in Spain showed a mean of 7.7±1.9 points in MD adherence, with 63.6% of students showing a low adherence.^20^ Similarly, another study also conducted in Spain found that 65.4% of students had a poor adherence to the MD.^21^ Students from the field of Social Sciences and Humanities showed the highest adherence to the MD (7.25±2.13), and despite having shown adequate NL (4.64±1.42) the students from Exact Sciences and Engineering showed the highest levels of NL (5.07±1.19). This may be due to a lack of proper time management, busy class schedules and stress experienced in this academic area, which consequently influences eating behaviours.^22^ It is important to highlight that in our sample, a notable positive trend (r=0.860, p=0.067) between NL and MD adherence was observed. Even though this result was not statistically significant, it remains relevant and suggests that NL plays a crucial role in promoting adherence to healthy dietary patterns like the MD.

In Portugal, 31.4% of the population aged >18 years presented low adherence to the MD, and this percentage was higher in males^23^; however, this trend was not observed in the present study, partly because the cut-off values were different; a score ≤3 was classified as low adherence, a score of 4 or 5 as moderate and a score of ≥6 was classified as high. Additionally, the mentioned study did not use the PREDIMED questionnaire, they used the definition of the *Mediterranean Diet Score* (*20–22*), with scores between 0 and 9. Gender differences were only seen for individual questions of the PREDIMED and not on the overall score, which may be explained by the fact that females tend to be more concerned about health, consequently tend to comply with the recommendations and have greater knowledge about healthy eating habits compared with males.

The favourable results observed regarding the use of olive oil as the main source of cooking fat, the frequency of sugary drink and pastry consumption, as well as the consumption of butter, margarine, or cream, vegetables, fish, legumes, and the preference for white meats are in line with the results obtained in other studies that assessed the same population and used similar methodologies.^19 21^ Furthermore, less favourable dietary patterns, such as low fruit, low nut intake, and high red meat consumption, were also observed in the study conducted by Cobo-Cuenca *et al* on university students.^21^

The frequency of daily fruit and vegetable consumption differs significantly from the WHO guidelines (400 g/day)^24^ and from the Mediterranean Food Wheel guidelines (3–5 servings/day).^7^ In Portugal, the average daily intake is 312 g/day, with 56% of the population not meeting the recommended doses.^23^ As mentioned above, during the first month of the pandemic, fruit (29.7%) and vegetable consumption (21.0%) increased across the Portuguese population.^25^ However, when compared with the first 12 months of the pandemic, intake of fruit decreased again, below the WHO recommendations.^26^

The high consumption of red meats (67.2%; n=733) observed in our sample is in line with records of national consumption (serving 100 g) (51.6%).^23^ It is worth noting that according to the International Agency for Research on Cancer, daily consumption of 100 g of red meat is associated with a 17% increased risk of colon cancer.^27^

Moreover, in the academic field of Health, the increased intake of nuts and the reduced consumption of red meats were the questions with the greatest strength in the final adherence score. According to the Directorate-General of Health, the Portuguese population has low adherence to the recommended consumption of nuts and dried fruits because there is a perception that these foods cause weight gain.^28^ It seems that for students from the Health field, this misperception does not apply because they tend to have a more adjusted knowledge regarding this subject, as shown by the results.

NL was adequate in 84.1% (n=433) of our participants. In concordance with these results, a study based on college students in Houston, a city in Texas, USA,^29^ also found 82% showed an adequate level of NL. It should be noted that in other studies lower results than these were found, and^28^ this difference is even more pronounced when compared with other studies based on a Portuguese population. Paiva *et al* evaluated a Portuguese adult population and observed that only 19% of the participants showed an adequate level of health literacy, also assessed using the NVS questionnaire.^30^ The discrepancy is not shocking, since individuals enrolled at a university finished high school, and therefore became more literate through the process.^31^ Additionally, the requirements to be admitted at the University of Lisbon are high compared with other universities in Portugal.

When NL (theoretical knowledge) is compared with adherence to MD (practice), it can be concluded that although students have adequate NL, they do not apply it to their lifestyle. This premise has also been verified by Miranda on medical students at the Faculty of Medicine of the University of Coimbra, Portugal, since they demonstrated adequate levels of knowledge but then did not transpose this knowledge into practice in their daily lives.^32^ Also in a cross-sectional study carried out in Greece, no significant correlation was found between adherence to the MD and NL.^33^ No association was found between the mean score of the NVS questionnaire (NL) and gender or academic field of study, which is in line with other existing literature.^18 34–36^

Nevertheless, students from Exact Sciences and Engineering showed a higher accuracy in answering questions related to calorie count and portion sizes. A possible explanation for this trend is that these students are more comfortable performing calculations and have higher numeracy skills. The NVS questionnaire emphasises many numerical aspects which can explain why students from this field had the highest levels of NL.^37^

A positive correlation was observed between the mother’s level of education and NL, indicating a link between higher maternal education and NL. Conversely, the father’s level of education showed no association with NL. Additionally, both the mother and father’s educational levels were positively correlated with MD adherence. These results emphasise the importance of parental education in shaping healthy eating behaviours, directly influencing the choices made by their children. If these eating behaviours are practised at home, children are more likely to adopt and maintain these habits throughout their adulthood. Interestingly, no significant relationships between parents’ educational level and NL were observed in a study conducted with students from the University of Beira Interior, Portugal.^38^ It is worth noting that in many families, mothers spend more time caring for their children and thus play a more significant role in nutrition and health decisions than fathers.^39^

There is limited information on the NL of the Portuguese university population using the NVS, highlighting the urge to increase knowledge in this area for better discussion. It is worth noting that during the pandemic, 44.5% of the Portuguese sought more information on health and healthcare on the internet.^6^ Younger people and those with higher educational levels find it easier to obtain information, which may have contributed to higher NL.^6^

Certainly, the present study has notable limitations that warrant discussion. Methodological aspects, particularly the online data collection method, might have influenced some of the results obtained. The size of the questionnaire and the fact that it was sent to the institutional email and not the personal email could have adversely affected the response rate. Furthermore, the voluntary nature of some questions led to skipping by some students, impacting the overall analysis. The inclusion of a larger number of female respondents has a direct impact on the statistical tests involving the variable ‘gender’; for a future study it would be ideal to include the same number of female and male participants. Nonetheless, this discrepancy reflects that females are more likely to answer health questionnaires, suggesting a higher level of concern among women. On the other hand, the strengths are that our variables were assessed using established and validated tools (PREDIMED and NVS); these tools support the reliability and credibility of our findings. For future studies, it would be beneficial to classify adherence to the MD into three categories; low, moderate and high, as this will provide a better understanding of MD adherence, allowing for a more comprehensive analysis and enabling more precise recommendations.

Most importantly, this study has contributed to the knowledge in this area. Its findings hold the potential to support and drive the development of public and community health initiatives in university settings. Given the pivotal role of individuals’ food education in their overall health, these insights become invaluable for shaping future health interventions and educational programmes.

## Data Availability

All data relevant to the study are included in the article or uploaded as supplementary information.
