# Supplementary material for: Mediterranean diet adherence and nutritional literacy: an observational cross-sectional study of the reality of university students in a COVID-19 pandemic context
Source: BMJ Nutr Prev Health. 2023 Dec 19;6(2):221–30. doi: 10.1136/bmjnph-2023-000659 (PMC10862292; doi:10.1136/bmjnph-2023-000659)
Supplement: Supplementary data [file bmjnph-2023-000659supp001.pdf]

Appendix

Appendix 1- Distribution of faculties by academic field of study

Academic field of study	Faculties
Health	Medical School<br>Faculty of Pharmacy<br>Faculty of Veterinary Medicine<br>Dental School
Social Sciences and Humanities	School of Arts and Humanities<br>School of Law<br>School of Social and Political Sciences<br>Institute of Education<br>Faculty of Psychology
Arts	School of Architecture<br>Faculty of Fine Arts
Natural Sciences	Faculty of Sciences<br>School of Agriculture<br>Institute of Geography and Spatial Planning<br>Faculty of human motricity
Exact Sciences and Engineering	Instituto Superior Técnico<br>School of Economics & Management
